# Implanted brain-computer interface functionality during nighttime in late-stage amyotrophic lateral sclerosis

**DOI:** 10.1038/s41598-026-44228-7

**Published:** 2026-03-18

**Authors:** Sacha Leinders, Erik J. Aarnoutse, Mariana P. Branco, Zac V. Freudenburg, Simon H. Geukes, Anouck Schippers, Malinda S.W. Verberne, Max A. van den Boom, Benny H. van der Vijgh, Nathan E. Crone, Timothy Denison, Nick F. Ramsey, Mariska J. Vansteensel

**Affiliations:** 1 Department of Neurology and Neurosurgery, UMC Utrecht Brain Center, University Medical Center, Utrecht, the Netherlands; 2 Department of Neurology, Johns Hopkins University School of Medicine, Baltimore, USA; 3 Medical Research Council Brain Network Dynamics Unit, Department of Clinical Neurosciences, University of Oxford, Nuffield, Oxford, UK; 4 Donders Institute for Brain, Cognition and Behavior, Radboud University, Nijmegen, The Netherlands

**Keywords:** Brain-Computer Interface, Electrocorticography, Home-use, Night, Circadian, Sleep, Engineering, Neurology, Neuroscience

## Abstract

Brain-computer interfaces (BCIs) hold promise as assistive communication technology for people with severe paralysis. Although such BCIs should be available 24/7, feasibility of nocturnal BCI use has not been investigated. Here, we addressed this question using data from an electrocorticography-BCI user with amyotrophic lateral sclerosis. We investigated nocturnal dynamics of neural signal features used for BCI control. Additionally, we assessed nocturnal performance of a decoder trained on daytime data, by quantifying the number of unintentional BCI activations at night. Finally, we developed a nightmode functionality and assessed its performance. Mean and variance of low and high frequency band power were significantly higher at night than during the day. When applied to night data, daytime decoders caused unintentional BCI activations in 100% of nights (245 unintended click-commands and 13 unintended caregiver-calls per hour). The specifically developed nightmode functionality, however, functioned error-free in 79% of nights over a period of ± 1.5 years, allowing the user to reliably call the caregiver. Reliable nighttime use of a BCI requires strategies to adjust to circadian and sleep-related signal changes. This demonstration of a reliable nightmode and its long-term use by an individual with amyotrophic lateral sclerosis underscores the importance of 24/7 BCI reliability.

**Supplementary Information:**

The online version contains supplementary material available at 10.1038/s41598-026-44228-7.

## Introduction

Neural disease or injury can lead to a state of almost complete paralysis while cognition is spared, so called locked-in syndrome (*LIS*^1,2^;. The ability to communicate is a determining factor for the quality of life in people with LIS^3,4^. Conventional augmentative and alternative communication (*AAC*) technology can assist people with LIS in communication but requires residual, reliable motor control^5^, which not all people with LIS are able to produce. These individuals may benefit from brain-computer interface (*BCI*) technology, which allows computer control based on brain signals^6–8^.

Several studies have shown that people with severe paralysis can use an implanted BCI for communication^9–19^. Moreover, first evidence of the feasibility and user benefit of unsupervised at-home use of implanted BCI systems has now been demonstrated^14,17,20^. Successful at-home use of a communication device by people with LIS, however, involves the ability to call caregivers and communicate needs not just during the day, but also at night. Importantly, research has shown that sleep affects brain activity in several regions^21,22^, including the sensorimotor cortex^23,24^, which constitutes the signal source of the vast majority of implanted BCIs.

We here used a unique dataset from a BCI user with late-stage amyotrophic lateral sclerosis (ALS) to investigate how BCI control signals in the sensorimotor cortex change at night. Second, we tested the nocturnal performance of BCI decoders that were optimized for daytime usage by applying them, offline, to night data. Finally, we developed and tested a dedicated nightmode functionality that allowed the BCI user to reliably generate a call-caregiver signal at night and assessed its performance in daily life settings over a period of ± 1.5 years.

## Methods

### Protocol approval, registration, and consent

The study was approved by the Medical Research Ethics Committee of the University Medical Center of Utrecht, the Netherlands. Research was conducted according to the Declaration of Helsinki (2013). The study was a registered clinical trial (*ClinicalTrials.gov: NCT02224469*). The participant gave informed consent using a procedure dedicated to people with severe communication impairments^17^.

### Participant information

The participant of this study is a woman (UNP1) diagnosed with ALS, who was enrolled in the Utrecht NeuroProsthesis clinical trial in September 2015, when she was in her fifties. At the time, she was quadriplegic and anarthric, and used an eye gaze device and eye blinks for communication. She received chronic tracheostomy invasive ventilation. Information about the participant’s cognitive and motor status over time has been provided in an earlier manuscript^20^. In November 2015, the participant was implanted with a BCI system, which enabled her to generate click-commands for communication. To produce a click-command, she attempted right hand fingertapping (sequential finger opposition) to generate brief changes in sensorimotor neural activity^17^. A second decoding algorithm^25^ detected a more sustained increase of sensorimotor cortex activity (> 7.6 s), generated by more long-term attempted fingertapping, which triggered a pop-up escape window (enabling quick caregiver calls, and an escape from software menus), or a system activation from daytime standby mode, depending on the AAC software context, henceforth referred to as *‘escape’* (Supplementary Figure S1).

### Data acquisition

The implant consisted of two electrocorticography (ECoG) electrode strips (Resume II ^®^, Medtronic; off-label use), with 4 electrodes each, placed subdurally over the hand knob region of the left sensorimotor cortex. Two other strips were implanted over the dorsolateral prefrontal cortex^26^. In October 2015, one strip from each brain region was connected to an amplifier-transmitter device (‘*implanted device’*; Activa ^®^ PC + s, Medtronic; off-label use), which was implanted subcutaneously under the left clavicle. In September 2020 (week 255 since implantation), the implanted device was replaced, to enable continued use of the system^20^. During this surgery, both sensorimotor cortex electrode strips were connected to the implanted device. The lead of the previously connected DLPFC strip was capped and left disconnected.

The implanted device wirelessly transmitted either raw potential data at 200 Hz (time domain) or frequency-decomposed data at 5 Hz (power domain)^17^ to an external antenna. The power domain mode required significantly less battery power than the time domain mode and was therefore always employed for independent at-home use, as well as for nocturnal measurements. Up to December 2021, the participant used a combination of low frequency band (*LFB*: set to 20 ± 2.5 Hz on the implanted device; effective bandwidth is broader) and high frequency band power (*HFB*: set to 80 ± 2.5 Hz; effective bandwidth is broader) from electrode pair E2E3 for daytime BCI control. As of December 2021, LFB power of E2E3 was combined with HFB power from electrode pair E10E12 to boost BCI performance, after it had gradually decreased^20^. Since only E2E3 data was used for BCI control at night (see *Nightmode Functionality Development* section) all data presented in this manuscript represents LFB and HFB power domain data from that electrode pair.

All data were recorded at the participant’s residence, either during research sessions or during independent at-home use. Night data and research session data that were used to develop decoding algorithms for night usage were recorded with the device that was implanted in 2015. All at-home BCI use data presented in this manuscript were recorded with the new device, between September 2020 and October 2022. The term ‘*recording file*’ refers to an individual data file (created when the BCI at-home use software was started and appended with neural data until the software was closed).

### Data selection procedure

Based on information from the participant’s caregivers regarding her daily schedule, we assigned files recorded between 12:00 h and 20:00 h to ‘day data’ and 0:00 h and 8:00 h to ‘night data’. Data recorded in the other periods were not analyzed because they typically involved extensive physical care. For analysis of day and night signals, we only considered days and subsequent nights with at least 6 h of data (not necessarily continuous recording files; Table 1), which are referred to as ‘Day-Night Datasets’. For calculating the nocturnal performance of the daytime BCI decoders, we used data from ‘Initial Night Measurements’, passive recordings during the night, conducted before device replacement (Table 1). Additionally, data recorded during daytime research sessions (Research Session Data; Table 1) was used to ensure the participant’s ability to reliably activate the nightmode functionality (i.e., few false negatives) during its development.

**Table 1:** Overview of different datasets. Datasets are presented in the same order as in the main text. Data recorded before week 255 was recorded with the first implanted device. Week numbers indicate weeks since implantation of the ECoG electrodes.

Dataset type:	Recorded during:	Number of datasets:	Used for:	Period:	Methods Section:
Day-Night Datasets: night data > 6 h duration with matching daytime dataset	At-home use^a^	209 day-night datasets	Analysis of day and night signals	Week 255–361	Data Analysis: Comparing Day and Night ECoG Signals
Initial Night Measurements	Passive, at-home data recordings^b^	17 nights	Quantification of nocturnal performance of decoders optimized for daytime use; Optimization of nightmode functionality.	Week 111–220	Data Analysis: Performance of Daytime BCI Decoder at Night
Research Session Data	Research sessions	14 repetitions of the research task (see the respective section)	Optimization of nightmode functionality	Week 220–244	Nightmode Functionality Development

^a^ All data marked with ‘at-home use’ was recorded during periods of active BCI usage.

^b^ The Initial Night Measurements were passive night recordings, during which the participant did not attempt to use the BCI.

### Data pre-processing pipeline

ECoG data was analyzed with MATLAB (The Mathworks, Inc., version 2021a). LFB and HFB power data, sampled every 200ms (5 Hz) with the implanted device, was smoothed temporally over the preceding 1.2 s (cf^17^. Subsequently, recording files were concatenated into one long data file and matched to a file with date and time information. Timepoints without brain data were filled with NaN (‘not-a-number’) values.

### Data analysis: comparing day and night ECoG signals

For analysis of the LFB and HFB power dynamics, we first established the normalized mean power of LFB and HFB signals for each day and night (Day-Night Datasets with > 6 h of data; Table 1). In addition, we established the variance in power during days and nights. Outliers (> 3 SDs from the mean) were removed from the data. Then the difference in mean power and variance between each day and subsequent night was calculated. Second order polynomials were fitted to every timeseries (12 in total; day, night and difference for power and variance, and for LB and HFB) to establish and account for potential signal deterioration over time. To compare day vs. night, we tested whether the time-average of the day vs. night difference series (detrended with the second-order polynomial fit) differed from zero using generalized least squares (GLS) with a continuous‑time AR(1) (Ornstein–Uhlenbeck/CAR(1)) correlation structure to adjust standard errors for serial correlation under irregular sampling. For the four day vs. night tests (power and variance for LFB and HFB), p‑values were adjusted using the Benjamini–Hochberg false discovery rate (FDR) procedure (q = 0.05).

### Data analysis: performance of daytime BCI decoder at night

We investigated how many unintentional click commands and escapes would have occurred during the night, if the decoding algorithms the participant used to produce these BCI commands during daytime at-home use of the BCI, were applied offline to nocturnal data. The nocturnal data used for this analysis was acquired in nights in which the participant did not yet use the BCI (Initial Night Measurements, yielding 86.2 h of data; Table 1). Therefore, any detected click-command and escape can be considered unintentional. We present an overview of the number of unintentional click-commands and escapes per hour. Notably, a formal analysis of unintentional click-commands and escapes for daytime data could not be made as the BCI was used during daytime, and the intentionality of each of the BCI commands was not logged. Yet, we have reported earlier that BCI performance was highly accurate for many years after implantation^20^.

### Nightmode functionality development

#### Data collection

Based on the results of the off-line evaluation of the performance of the daytime BCI decoders at night, we concluded that a dedicated approach was needed to provide the participant with the option to leave the BCI system on during sleep with minimal false positive activations, while giving her the option to call a caregiver at night when needed. An additional requirement was that the system should not depend on visual stimuli, because the participant’s eyes were kept closed at night with medical tape, to prevent uncontrolled opening and subsequent drying of the cornea. Data recorded during seventeen nights in the period of Dec 2017 to Jan 2020 (weeks 111–220) were used to investigate the spontaneous occurrence of decoder activations with the aim to develop a nightmode functionality that would produce a minimal number of false positives (Initial Night Measurements; Table 1). Additionally, data recorded during daytime research sessions (Research Session Data; Table 1) was used to ensure the participant’s ability to reliably activate the nightmode functionality (i.e., few false negatives). In these research sessions, the participant tested the activation of the nightmode functionality by performing different mental strategies. Several strategies proved unsuccessful. An overview of the attempted strategies is presented in the Supplementary Results and Supplementary Table S1 and S2. Feasibility of the strategy that was eventually implemented and utilized was tested in 14 repetitions of a research task (Research Session Data; recorded with the first implanted device in weeks 220–244 across five research sessions; see Table 1).

#### Nightmode functionality solution

The implemented nightmode functionality employed a sequence of correctly timed *‘nightmode events’* that were based on the LFB rebound. Nightmode events were defined by a strong LFB increase that occurred upon the termination of attempted movement. To cue the nightmode events, the participant used the timing of her ventilation machine, which was usually set to 15 cycles per minute (*CPM*) and was sometimes adjusted to 14 or 16 CPM. The mental strategy the participant executed to call her caregiver at night was attempted hand movement during one 4-second ventilation cycle (when using 15 CPM), and rest during the next ventilation cycle. This sequence was repeated, leading to a LFB rebound approximately every 8 seconds. An individual nightmode event was registered when the normalized LFB signal passed an empirically determined threshold of −0.4 for 2 seconds. Normalization was based on a 30-second calibration period that was completed before switching on the nightmode. During the 30-second calibration, the participant attempted three hand movements. The nightmode event interval parameter was set to 6–10 sec (centered around the 8-second duration of two ventilation cycles), allowing for timing deviations and changes in the ventilation settings from 14–16 CPM. Other algorithm parameters were the required number of cycles, the rate, and the variance cutoff (Supplementary Table S3). The algorithm applied a sliding window to the preceding x seconds, with x being the number of cycles multiplied by the duration of one active-rest cycle (8 sec when using 15 CPM). The number of correctly timed nightmode events in the window was counted. When all requirements were met, the BCI system was activated, enabling the use thereof to call the caregiver (more info in *Nightmode User Interface*).

#### Nightmode user interface

The participant was able to set the system to enter nightmode with regular click commands. Alternatively, a caregiver could switch on the nightmode by using the touch screen. Immediately after the 30-second calibration and switching on the nightmode, the participant often performed a test BCI activation, and repeated the 30-second calibration if the test was unsuccessful. Supplementary Figure S2 shows all user interface screens of the nightmode and its logic.

### Data analysis: nightmode performance

We present information on frequency of at-home use and performance metrics of the nightmode. Performance data was collected by caregivers on night-duty, who were asked to assess whether a BCI activation at night was intentional or not (true/false positive), by asking the participant each time she had activated the nightmode. In addition, caregivers asked in the morning whether the participant tried to activate the system and if that was successful (true/false negative). This information was transmitted to the research team by (initially) daily phone calls, and (later) a written log kept by the caregiving team. Finally, based on qualitative reports, we present several real-life examples of reasons for using the nightmode to call a caregiver, and user satisfaction.

## Results

### Comparing day and night ECoG signals

**Fig. 1:**
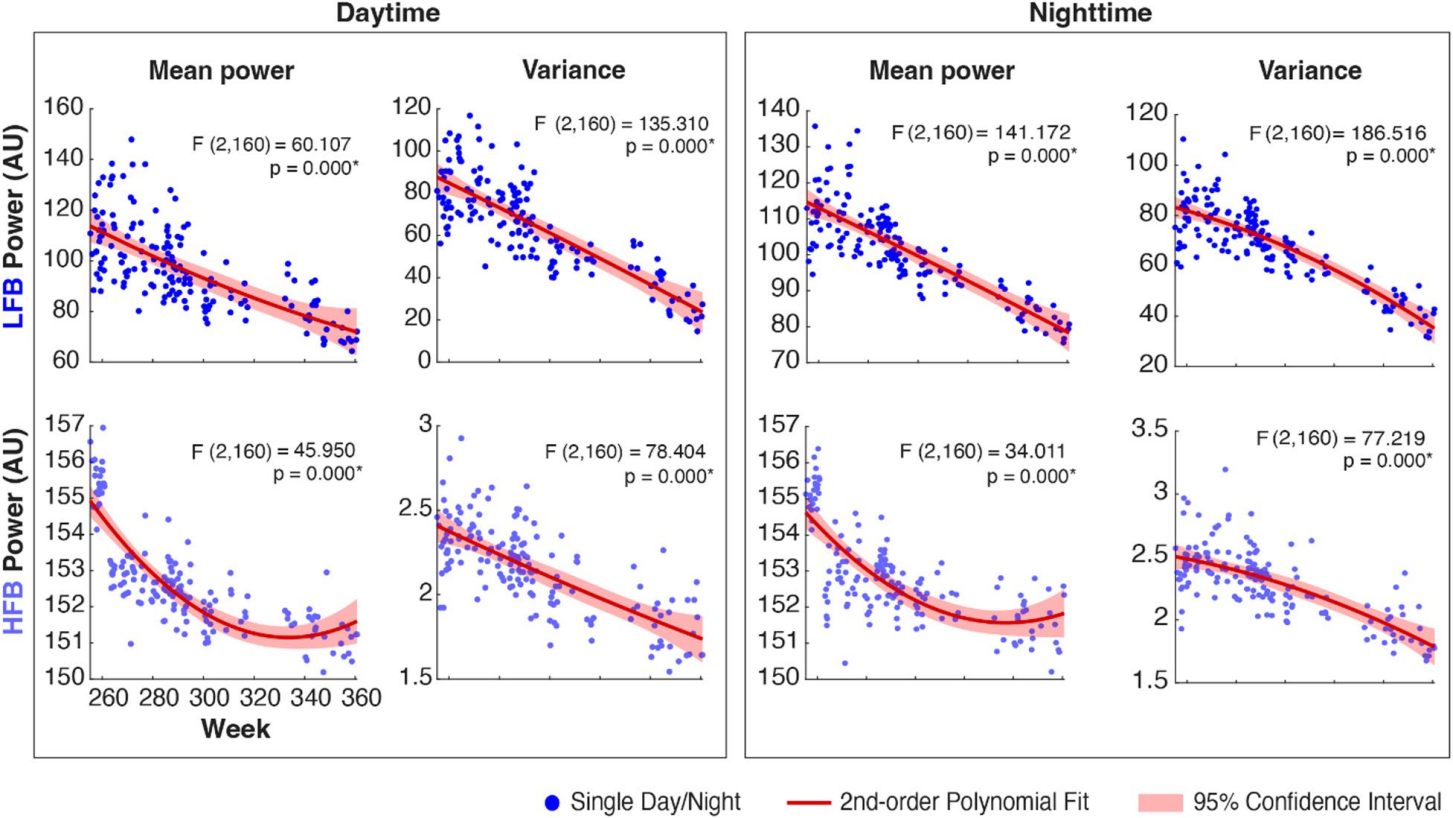
Mean power and variance of daytime and nighttime HFB and LFB signals. Each blue dot represents a single day or night. The fitted second-order polynomial is represented by the red curve, and its surrounding pink shaded area is the 95% confidence interval of the fit. The F- and p-values correspond to a test of the null hypothesis (H₀) that there is no change over time. All the displayed degrees of freedom and confidence intervals are established while accounting for serial correlations (AR(1)).

**Fig. 2:**
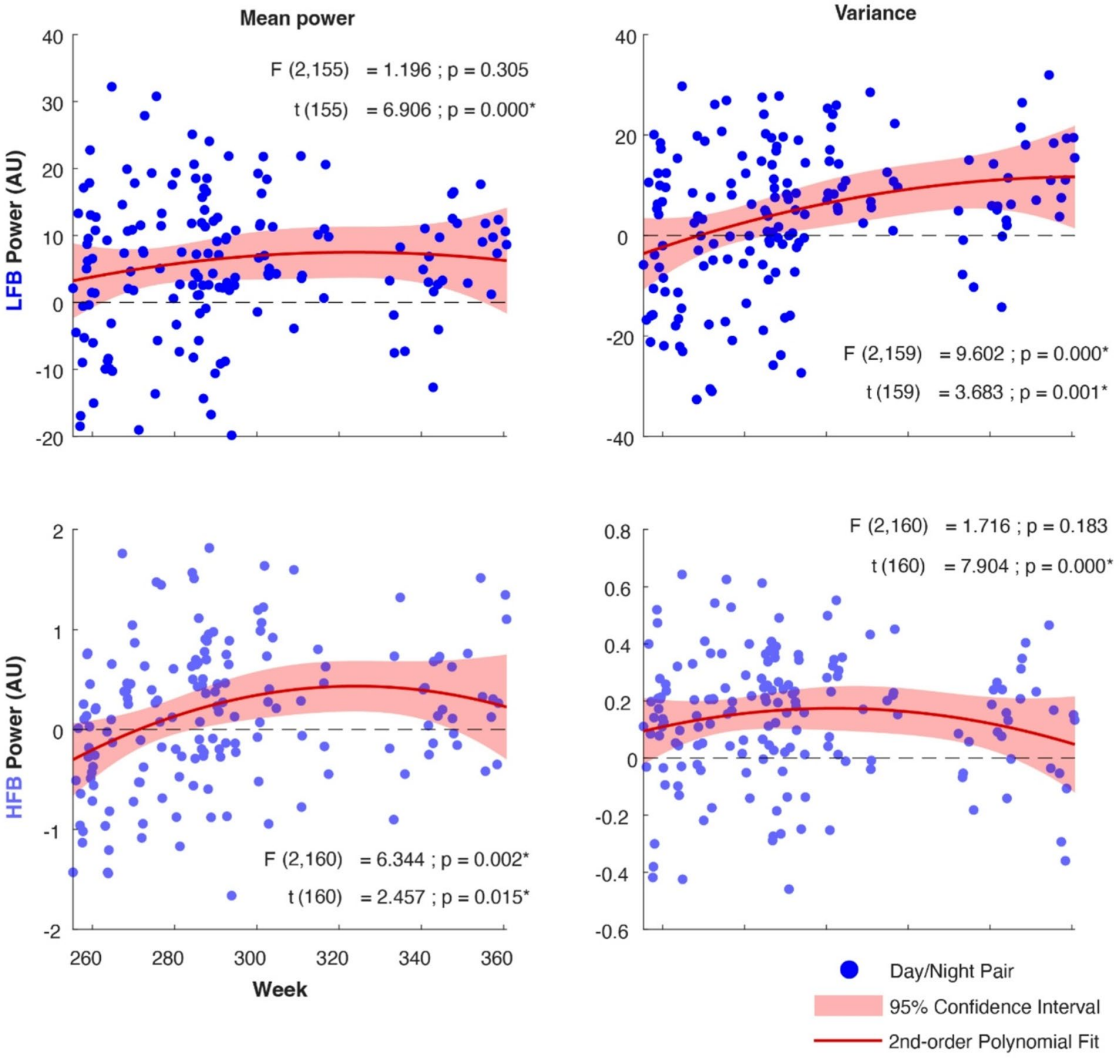
Mean power and variance difference between daytime and nighttime for LFB and HFB signals. Each blue dot represents the difference between a single day and subsequent night. The fitted second-order polynomial is represented by the red curve, and its surrounding pink shaded area is the 95% confidence interval of the fit. The t and p-value show the test-results for the difference between night and day across all weeks (alpha < 0.05; FDR corrected for the 4 tests). The F- and p-values correspond to a test of the null hypothesis (H₀) that there is no change over time. All displayed degrees of freedom and confidence intervals were established while accounting for serial correlations (AR(1)).

The LFB and HFB mean power and variance during the day and during the night all revealed significant decreases over time (Day-Night Datasets; Table 1; Fig. 1). Testing the difference between day and night revealed larger power and variance during nighttime than during daytime for both LFB and HFB signals (Fig. 2). This difference significantly increased over time for LFB variance and for HFB power (Fig. 2).

### Performance of daytime BCI decoder at night

**Fig. 3:**
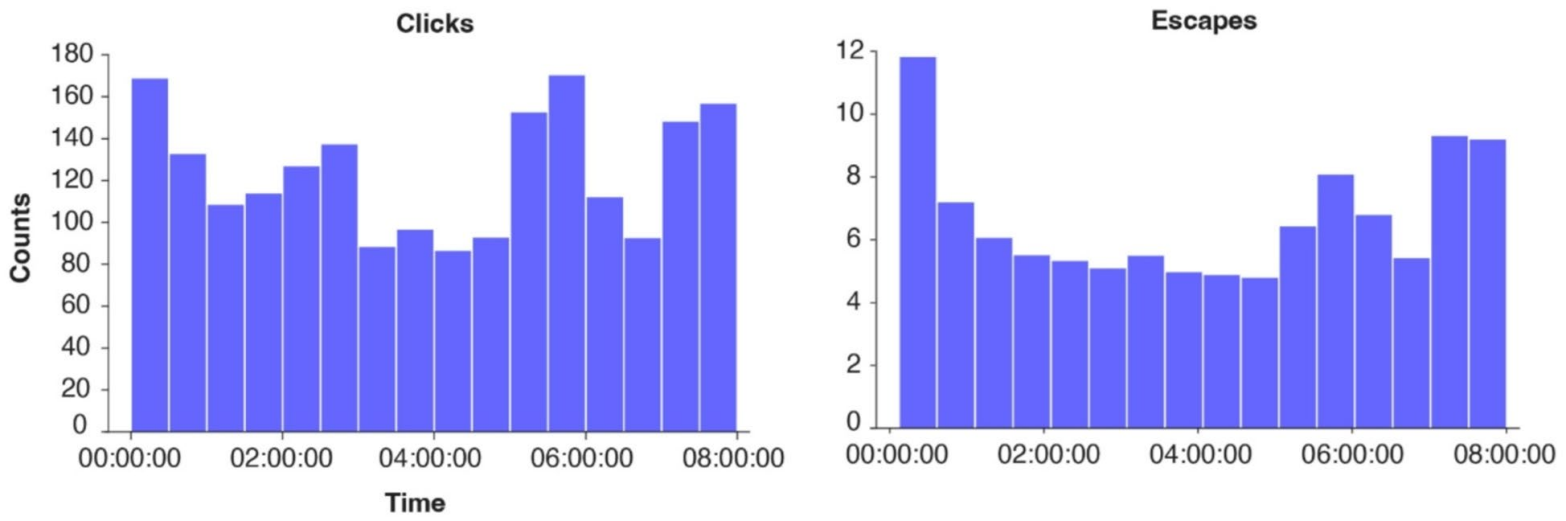
Distribution of unintentional clicks and escapes. The number of unintentional clicks (left) and escapes (right) plotted in 30-minute blocks, calculated on the Initial Night Measurements (Table 1) performed before installing the nightmode (86.2 h of data recorded across 17 nights). Each bar indicates 30 min of the period between 0:00 h − 8:00 h, which was considered night, as per caregiver information.

Offline application of the daytime decoders for click-commands and escapes on data that was acquired during the passive Initial Night Measurements (86.2 h over 17 nights; Table 1) in a period where the BCI was not yet used during the night, resulted in 245 unintentional clicks and 13 unintentional escapes per hour during the night (Fig. 3). All of the 17 nights contained errors.

### Nightmode functionality and performance

#### Nightmode parameters

**Fig. 4:**
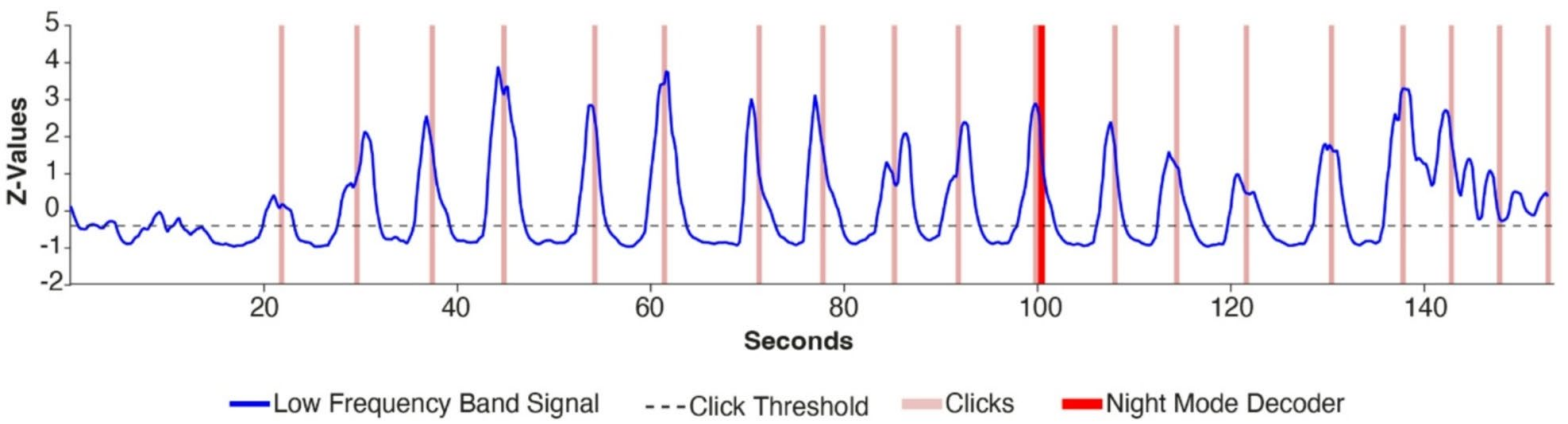
Nightmode sequence example. LFB power recorded during a research task in which the participant was asked to follow the block design of her ventilation machine (4 s of attempted hand movement, 4 s of rest). Besides an auditory start (at 20 s) and stop cue (at 140 s) no other task cues were provided. In this case, the nightmode functionality was activated after 11 nightmode events even though it was set to 12 cycles. This happened because the rate was set to 0.01, allowing for one false negative at any point in the sequence. The absence of any false negative nightmode event between cycles effectively activated the nightmode functionality one cycle sooner.

**Fig. 5:**
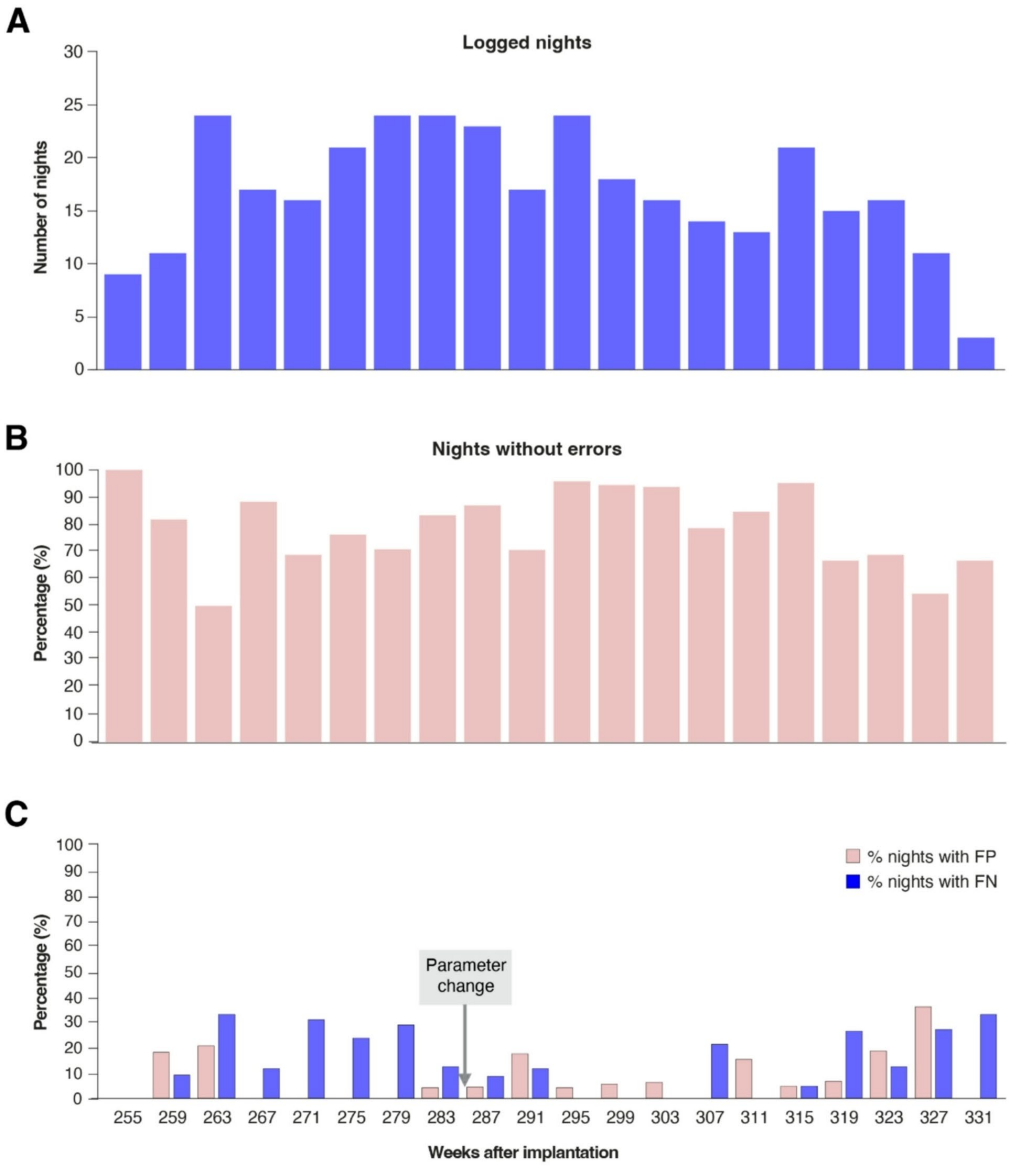
Nightmode data overview and performance. X-axes indicate week number since electrode implantation, with each week number representing the start of a 4 week period. A: Number of nights per 4-week period for which performance was logged. B: The percentage of nights without any false positives (FP) or false negatives (FN). C: The percentage of nights that contained 1 or more false positive (FP) and 1 or more false negatives (FN). Notably, the ‘number of cycles’ parameter (see Supplementary Table S3) was changed in week 284 from 12 to 11 in an attempt to improve performance of the nightmode functionality (decreasing the number of FNs). In week 286 it was changed from 11 to 10, after which two false positives occurred in the subsequent night. The number of cycles was then reverted to 11, as the participant preferred low FPs over low FNs. This parameter change led to 9% and 14% of nights with FPs and FNs, respectively in the period from April 2021 until March 2022 (week 283 – week 334, when the last nights were logged).

The parameters for the nightmode functionality that was implemented and utilized are shown in Supplementary Table S3. With these parameters, the system was activated as intended (true positive) in 13 out of 14 research task repetitions acquired during research sessions (Research Session Data; Table 1) and no false positives were present in the Initial Night Measurements. The only parameter that was adjusted in the period the participant used the nightmode at home was the number of cycles (see Figure legend of Fig. 5 for details). An example of the pattern of signal changes required to activate the system and call the caregiver at night, recorded during a research session, is shown in Fig. 4.

#### Nightmode use and performance

Between September 2020 and October 2022, the participant used the BCI in 494 nights. Between week 255 and week 334, nightmode performance was logged for 337 nights (Fig. 5A). The number of logged nights decreased in the final months, when overall use of the system became more erratic and communication at night became more difficult^20^. As a result, caregivers could no longer determine if a nocturnal caregiver call was intended. Caregivers stopped filling out the performance logs from April 2022 onward. Nevertheless, the nightmode continued to be in use on the participant’s request until early October 2022 (week 361). In consultation with the participant and her caregivers it was then decided to no longer use the nightmode, due to unreliable performance and communication.

The nightmode functioned without errors in 79% of all logged nights (Fig. 5B). In 33% of all logged nights, only true negatives occurred (i.e., no nocturnal caregiver calls were attempted and no false positive occurred). 58% of nights contained true positive nocturnal caregiver calls and on average there were 2.3 caregiver calls in those nights. 15% of all nights contained false negatives (on average 2.4 in those nights) and false positive caregiver calls occurred in 8% of nights (once every ± 12 nights; Fig. 5C). Individual nights could contain combinations of false and true, and positive and negative events.

Based on qualitative reports kept by caregivers, the participant used the nightmode to call caregivers mostly for care-related requests, such as lung suctioning or medication. The participant reported satisfaction with the nightmode on multiple occasions to researchers during research sessions.

## Discussion

For real-life use, a BCI communication device should be available and function reliably 24/7. We show, in a person with late-stage ALS who had an implanted BCI system, that decoders that performed well during daytime generated high rates of false positive detections of BCI actions during the night, related to different nocturnal signal characteristics, which in effect would render the system useless at night if not accounted for. We also present a nightmode solution that allowed the user to activate the BCI system and call caregivers reliably at night, with few false positive activations.

The most noticeable observations when comparing day and night signals were that LFB (10–30 Hz) and HFB (65–95 Hz) power and variance were overall higher during the night than during the day. This result contradicts that of Cantero and coworkers^27^ who found higher gamma variance during wakefulness than during sleep in four able-bodied epilepsy patients. This discrepancy may be explained by the motor impairment of the participant of this study, and the associated lack of activation of the sensorimotor cortex. In the period of study (i.e., after week 255 post-implantation), the participant only moved when communicating yes-no with the corner of her mouth. In addition, cortical activity associated with BCI use and passive movement of limbs can be expected to occur far less frequently than the almost continuous activation expected due to movement and sensory activity in able-bodied people, even when they are bedridden such as in^27^.

The nature of the variability in HFB and LFB power at night is currently unclear. One potential explanation for the variability is that the participant sometimes napped during the day. Naps of more than 30 min long have been suggested to affect nighttime sleep^28^. No logs on naps or actual nocturnal sleep were kept (due to caregiver workload constraints). Second, it cannot be excluded that different levels of passive movements, such as those related to caregiving, account for differences of LFB and HFB power across different day/night combinations. Third, different sleep-stages (NREM/REM) are known to be associated with different neural signal characteristics^22,29,30^. Whereas the current dataset did not allow to distinguish between sleep-stages, different distributions thereof between days/weeks/months may be associated with different power and variance. Finally, research has shown that sleep is often affected in people with ALS^31,32^. Specifically, sleep efficiency is often decreased and wake time after sleep is increased. In our study, differences in sleep quantity and quality between nights may have led to variability in HFB and LFB power and variance. Further research will be needed to gain a full understanding of the effects of sleep and any ALS-related changes therein, on BCI control signals in the sensorimotor cortex.

Unintended BCI classifier activations should be kept to a minimum at all times but especially during the night, since they could interrupt the sleep of both the user and caregivers. We found that many false positive events were produced when classifier parameters optimized for daytime usage were applied to night data. The data used for this offline analysis was collected during the period in which the participant was not yet using the BCI at night. Therefore, all clicks and escapes that were detected by applying daytime decoders on nocturnal data can be considered unintentional, false positive BCI activations. Given that the BCI was used during the day, checking the same decoders for false positive generation during daytime home use could not be done, as ground truth information about the intentionality of click-commands and escapes produced would be lacking. Importantly, however, whereas BCI performance in this participant eventually declined due to ALS progression^20^, daytime decoders were highly accurate in the period the data for this assessment was acquired (~ year 2–4 after implantation; cf. Figure 1C of^20^. We therefore conclude that the erroneous decoding of clicks and escapes during nighttime was not caused simply by a lack of parameter optimization, but by spontaneous changes in the nocturnal signals for which the daytime decoder was not optimized.

The nightmode functionality was used by the participant for over two years. It allowed her to call caregivers when needing attention at night, The relatively long decision period of the nightmode algorithm (approximately 1.5 min) seems impractical at first glance, but was considered usable by the participant, particularly given the absence of any other option. The eventual decline in the logging and use of the nightmode coincided with the overall deterioration in BCI performance of this participant reported earlier^20^. We have ascribed this decline to progressive ALS, which is known to affect upper motor neurons in the sensorimotor cortex and may additionally affect cognitive abilities and attention^33^. The relative success of this nightmode underscores the importance of applying an iterative and user-centered design approach in these types of clinical trials.

The impact of daytime decoding errors on daily use of the BCI system is limited, provided they occur sporadically. At night on the other hand, each false positive caregiver call will wake the user and their caregiver, negatively affecting sleep quality of both. In addition, an inability to call the caregiver when attention is needed (false negative) may leave the user in discomfort or pain. The performance requirements for BCI use at night can therefore be considered more strict than those for daytime usage. For example, up to December 2021 (week 318) 1 in 12 nights contained an unintended caregiver call, which was deemed acceptable by the participant and her care team. However, when the number of false positives increased (e.g. after a parameter change in April 2021 (week 286, see Fig. 5) to 2 false positives in one night) or when it was no longer possible to ascertain the validity of the system, caregivers were reluctant to rely on the system. A second requirement is the need for unobtrusive cues instead of visual or auditory cues that may disrupt sleep or that may be otherwise unusable for particular individuals. The current solution relied on the pace of the ventilation machine, which turned out to work well for the participant. Similar methods of using unobtrusive timing cues for generating precisely timed changes in neural signals that are unlikely to occur spontaneously during sleep may work for other users of similar BCI systems, but parameters will most likely require customization. The current BCI system provided a one-dimensional control signal. The fact that the participant required at least 11 active-rest cycles to minimize false positives at night underscores the large variability of sensorimotor brain signals. Other ECoG-based BCI systems that employ multidimensional control signals (e.g^10,13,34^) or BCI systems using intracranial micro-electrode arrays (e.g^15,16,18,19^) are being tested. It is likely that also these more advanced BCI systems, if they were to be implemented in the daily life of the people they are meant to serve, will have to be adjusted to cope with circadian or sleep-related signal changes in the neural signals. Indeed^35^, reported false positive decoded events (2-D cursor movements) in data recorded during sleep in a BCI participant with a multi-electrode array in the sensorimotor cortex. Further research will be needed to clarify whether or not specific neural signal features are more resistant than others to the effects of day and night, and to develop strategies to overcome these differences.

In the nightmode, activating the system was accomplished through an intentional mental strategy. An automatic classifier that detects when someone is asleep or awake would be preferable as it does not require any active input from the user, but this requires reliable, automatic sleep-stage detection from intracranial signals, which the current dataset was unable to provide. Such feature requires more research, and may benefit from recordings of brain regions other than those used for direct BCI control.

In conclusion, we report on day-night differences in ECoG signals measured in an implanted BCI user with late-stage ALS. We showed that, in this study participant, LFB and HFB sensorimotor signals used for BCI control were subject to spontaneous signal perturbations during the night, and that applying BCI decoder parameters optimized for daytime use to night data caused many unintended decoder activations. We present for the first time a nightmode solution that allowed a BCI user to reliably call a caregiver at night. Providing a BCI system that can cope with circadian and sleep-related brain signal changes is the difference between a BCI system that is available 24/7 and a BCI system that is usable 24/7.

## Supplementary Information

Below is the link to the electronic supplementary material.

Supplementary Material 1

## Data Availability

Reasonable requests for sharing of the data and code can be made to the corresponding author.
